# Establishment of a Near-Infrared Spectroscopy-Based Screening Framework for Key Quality Indicators of Cassava Varieties

**DOI:** 10.3390/foods15173139

**Published:** 2026-09-04

**Authors:** Huimin Guo, Wenting Wang, Chenghong Liu, Shengyuan Guo, Wenjun Ou, Fei Gao, Kaimian Li, Lizhen Zhang, Guixing Ren

**Affiliations:** 1 Biotechnology Research Institute, Shanghai Academy of Agricultural Sciences, Shanghai 201106, China; 2 School of Life Science, Shanxi University, Taiyuan 030006, China; 3 Tropical Crops Genetic Resources Institute, Chinese Academy of Tropical Agricultural Sciences, Haikou 571101, China

**Keywords:** cassava, near-infrared spectroscopy, quality prediction, model validation

## Abstract

Cassava is the sixth most important food crop globally, valued for its high starch accumulation in tuberous roots, which supports diverse applications in food processing and industrial production. Quality evaluation is vital for its utilization, yet rapid and efficient assessment methods remain underexplored. In this study, 67 cassava varieties were collected to assess their nutritional quality. Near-infrared spectroscopy (NIRS) in the wavenumber range of 11,000–4000 cm^−1^ was applied to predict key quality traits of cassava flour from different varieties. Combined with partial least squares (PLS), principal component analysis (PCA), and internal cross-validation, a preliminary NIRS-based screening framework for cassava flour quality indicators was established. The protein and moisture models achieved an excellent quantitative prediction performance ($R_{cv}^{2}$ > 0.82, RPD > 4.0), qualifying them as reliable tools for routine analysis. In contrast, the models for starch, amylose, and amylopectin showed substantial overfitting with low cross-validation accuracies ($R_{cv}^{2}$ = 0.19–0.38), restricting their use to only preliminary screening. The fat model performed poorly ($R_{cv}^{2}$ = 0.16, RPD = 1.08), rendering it unsuitable for any quantitative or screening application. This study provides an exploratory screening framework for rapid multi-index evaluation of cassava flour quality. However, the marked variability in predictive performance across constituents highlights the critical need for external validation to improve model robustness.

## 1. Introduction

Cassava (*Manihot* esculenta Crantz) is an important root crop in tropical and subtropical regions, and it serves as a staple food for over one billion people worldwide [1]. Cassava originated in South American countries and gradually spread to Asia and Africa after the 16th century [2]. According to the Food and Agriculture Organization (FAO) of the United Nations, the global cassava production exceeded 300 million tons in 2022, which demonstrates the increasing global demand for cassava. Therefore, cassava is crucial for ensuring food security, not only because of its high adaptability to drought and low-fertility soils, but more importantly because it has made important contributions to alleviating poverty and hunger. It is also one of the most important staple foods in the global tropics (especially in Africa) [3]. Cassava roots typically contain 80–90% starch, and due to their unique thickening properties and high starch content, they are widely used in both the food and non-food industries [4]. The chemical composition of cassava starch is typically closely related to its cultivar, while the traditional reliance on stem segment vegetative propagation is prone to causing germplasm degradation, which in turn affects starch quality [5]. However, the functional characteristics and quality of cassava are crucial for breeding and market application development. Currently, there are hundreds of cassava varieties on the market, but the quality varies widely, and there is no unified evaluation standard. Traditional cassava quality detection mainly includes the detection of food safety (e.g., cyanide content) and commodity quality (e.g., starch content, variety authenticity) [6]. Hence, a rapid method capable of concurrently assessing multiple components is urgently required.

Near-infrared spectroscopy (NIRS) is a rapid, efficient, convenient, and non-destructive analytical technique. Compared to traditional chemical methods, it has gained significant attention in food quality analysis due to its distinct advantages, such as no complex sample preparation, no chemical reagents, no pollution, environmental friendliness, and ease of operation [7,8]. The modern application of NIRS can be traced back to the late 1950s, when Norris used this technology for rapid detection of crop quality [9]. As an indirect analytical technique, NIRS does not directly measure the substance itself, but analyzes the response of the substance to electromagnetic radiation in the 12,500–4000 cm^−1^ (i.e., 800–2500 nm) band, according to the position, shape and intensity of the formed spectrum. The characteristics are used to infer its composition and content [10,11]. In recent years, Khamsopha et al. have employed near-infrared hyperspectral imaging to quantitatively predict the adulteration of cassava starch [12]. Studies have shown that NIRS could not only measure basic components, but also predict the functional properties of cassava granules (water absorption capacity, swelling power, bulk density, and dispersibility), which are used to assess the food quality of cassava granule products [13]. However, NIRS detection models for assessing the quality of different cassava varieties remain relatively scarce at present. Most focus on predicting single or limited components, resulting in limitations during practical application. The robustness and transferability of these models are often limited, because the accuracy of their prediction models is highly dependent on the representativeness, diversity and quantity of the samples used to construct the models, while the calibration samples rarely cover a wide range of varieties, origin and growth conditions. Therefore, there is currently a lack of a comprehensive and reliable evaluation system that could simultaneously predict multiple key quality traits in diversified cassava germplasm resources, which is crucial for variety breeding and product development.

It is hypothesized that NIRS, combined with basic quality data from diverse cassava varieties, enables the construction of a rapid screening model that accurately predicts key quality indicators. To test this hypothesis, 67 cassava samples were collected and analyzed for basic nutritional components and color traits. Based on the collected spectral information, a quantitative prediction model for key quality indicators was established using PLS. In addition, the relationship between these qualities of cassava flour was further determined by correlation analysis and principal component analysis (PCA). This study provides technical references for the rapid evaluation of cassava quality and indicates the improvement direction for subsequent model optimization.

## 2. Materials and Methods

### 2.1. Sample Collection and Preparation

A total of 67 cassava varieties (Table S1) were collected from the cassava planting resource nursery of Danzhou Agricultural Base, Institute of Tropical Crop Genetic Resources, Chinese Academy of Tropical Agricultural Sciences. For each variety, three plants were randomly selected from the same field test area, and all tubers from each plant were harvested. All varieties were planted under uniform field conditions with consistent soil type, irrigation and agronomic management, and harvested after a 12-month growth cycle. The harvested cassava tubers were peeled, cut into small pieces, and dried at 50 °C. The dried pieces were ground through a 60-mesh sieve, and the obtained powder was sealed in plastic bags and stored at 4 °C. The analytical grade copper sulfate, potassium sulfate, glacial acetic acid and calcium chloride trihydrates were obtained from Hengxing Chemical Reagent Manufacturing Co., Ltd. (Tianjin, China). The analytical grade hydrochloric acid, sulfuric acid and hydrogen peroxide were obtained from Chinese Medicine Chemical Reagent Co., Ltd. (Shanghai, China).

### 2.2. Measurement of Color Characteristics

All cassava flour color measurements were performed using the CR-400/410 colorimeter (Konica Minolta, Inc., Osaka, Japan). All our tests were conducted in triplicate. Before the test, the instrument was calibrated using a white standard plate (L* = 92.34, a* = 0.13, b* = 3.41). The overall color difference (ΔE*) was determined using Equation (1).

(1) $$∆E=\sqrt{∆L^{*2}+∆a^{*2}+∆b^{*2}}$$

where L* is the lightness (0 for black and 100 for white), a* is redness (+) to greenness (−), and b* is yellowness (+) to blueness (−).

### 2.3. Nutritional Quality Testing

The primary nutritional components are moisture, protein, fat, and starch-related content. Moisture content was determined according to GB5009.3-2016. The protein content was detected according to the Kjeldahl method in GB5009.5-2016. The principle was to completely convert organic nitrogen into inorganic ammonium salt by strong acid and catalyst, and then determine the amount of ammonium salt, to calculate the total nitrogen content in the sample. The fat content is detected according to the soxhlet extraction method in GB5009.6-2016 [8]. The principle is to use the solvent reflux and siphon action to continuously and automatically extract the soluble components in the solid material for multiple cycles, thereby achieving efficient and thorough separation and enrichment. The total starch content and amylopectin content were determined using the total starch kit and amylose/amylopectin kit (Megazyme International Ireland Ltd., Wicklow, Ireland; total starch assay kit catalog number: K-TSTA-100A; amylose/amylopectin assay kit catalog number: K-AMYL). The difference between total starch and amylopectin is the content of amylose. For all tests each sample was measured three times independently, and the arithmetic mean value was taken as the result.

### 2.4. NIRS Acquisition

Before the experiment begins, preheat the instrument for 30 min while maintaining a constant experimental environment at 25 °C and 60% relative humidity [14]. All cassava flour samples were spectroscopically analyzed using an Antaris II FT-NIR spectrometer (Thermo Electron Co., Waltham, MA, USA) with a light-blocking sample cover in an opaque environment. Additionally, we collected empty rotary quartz sample cups as background samples. The NIRS of cassava flour was performed 32 times in the wavenumber range of 11,000–4000 cm^−1^, with a resolution of 8 cm^−1^. Each cassava flour sample was scanned three times, and the three repeated measured spectra were averaged to obtain a representative spectrum of each sample, which was then used for subsequent model calibration. In this study, TQ Analyst 9.0 software was used to analyze near-infrared spectroscopy (NIRS) spectra and model construction.

### 2.5. Establishment of NIRS Model

#### 2.5.1. Removal of Outliers

Before spectral preprocessing, a combination of Mahalanobis distance and chi-square test was used to remove all outliers in the original spectrum to ensure the standardization of model establishment. Any sample with a Mahalanobis distance exceeding 11.07 was considered a spectral outlier and removed. The number of spectral outliers removed was 3. The elimination of these three samples was completed before any spectral preprocessing and model establishment. Mahalanobis distance outlier detection was only used for spectrally abnormal samples, not nutrient extreme values. At the same time, prior to initiating data analysis, we standardized all obtained indicator concentrations.

#### 2.5.2. Spectra Pretreatment

During the spectral data acquisition process, cassava flour is susceptible to influences from the surrounding environment, equipment stability, its own particle size, uniformity, and other interfering factors [15]. Therefore, to minimize various interfering information and ensure the accuracy and stability of the model analysis, we employ multiple techniques to preprocess the NIRS diffuse reflectance spectral data. These techniques mainly include multiplicative signal correction (MSC) and standard normal variate (SNV). Meanwhile, to avoid spectral shift or drift, we employ first-order and second-order derivatives for processing, aiming to eliminate the offset baseline and background interference. In addition, the Savitzky–Golay filter (SGF) and Norris derivative filter (NDF) demonstrate denoising and smoothing effects on spectral data to improve the spectral signal-to-noise ratio [16]. In this study, the above methods were used for comprehensive pretreatment. Through systematic combination screening procedures, the best spectral pretreatment method was selected to determine the most effective method. For each combination, the PLS model was constructed and evaluated using 10-fold cross-validation RPD (residual predictive deviation) as the ranking criterion.

#### 2.5.3. Choice of Modeling Method

PLS, as the most used calibration method, was employed in this study. PLS is widely used in spectral analysis, as it can compress useful information within spectral bands into a low number of optimal latent variables (LVs), effectively addressing regression problems where the number of variables significantly exceeds the sample size [17,18]. In addition, the LVs of each PLS model are based on the cross-validation root mean square error (RMSECV) as the main selection criterion. Table 1 lists the final number of latent variables selected for each component, ranging from 6 to 10, with most models using 8–10 latent variables. Although these values are small relative to the sample size (n = 67), we acknowledge that there is still a certain risk of overfitting when using up to 10 LVs, especially in the case of weak chemical signals or ones masked by dominant matrix components (such as fat and starch-related components). Compared to the traditional multiple linear regression model, the PLS regression model is more effective at distinguishing system information from noise. PLS has been widely used for predicting potato varieties, corn varieties, and cereal varieties [19,20].

#### 2.5.4. Validation of the Model

In the process of establishing the NIRS model, it is usually necessary to verify the model to confirm the fitting degree of the model. At the same time, relevant data are needed to further verify the accuracy of the model. To evaluate the predictive performance and robustness of the model, this study employs K-fold cross-validation. Specifically, 67 samples were grouped according to their variety attribution to ensure that all replicates of the same variety were divided into the same fold during the cross-validation process. Subsequently, K = 10-fold cross-validation was performed. Each subset was successively treated as the validation set, while the remaining K-1 subsets served as the training set for model training and prediction. This process is repeated K times to ensure that each sample is predicted once. We used the calibration coefficient of determination ($R_{c}^{2}$), calibration root mean square error (RMSEC), cross-validation coefficient of determination ($R_{cv}^{2}$), cross-validation root mean square error (RMSECV), and residual prediction deviation (RPD) values to evaluate the predictive ability of the NIRS model. The higher the RPD value, the higher the accuracy and predictive ability of the model. Generally speaking, RPD > 2.5 indicates good predictive ability, 2.0–2.5 approximates quantitative screening, and <2.0 is not recommended for prediction [21].

### 2.6. Statistical Analysis

The data were expressed as the mean ± standard deviation (Mean ± S.D.) and analysis was performed by one-way ANOVA using IBM SPSS statistical software version 22.0. Statistical graphs and principal component analysis (PCA) were performed using Origin 2024 (Origin Lab Inc., Northampton, MA, USA). *p* < 0.05 was significant.

## 3. Results

### 3.1. Color Characteristics

**Figure 1:**
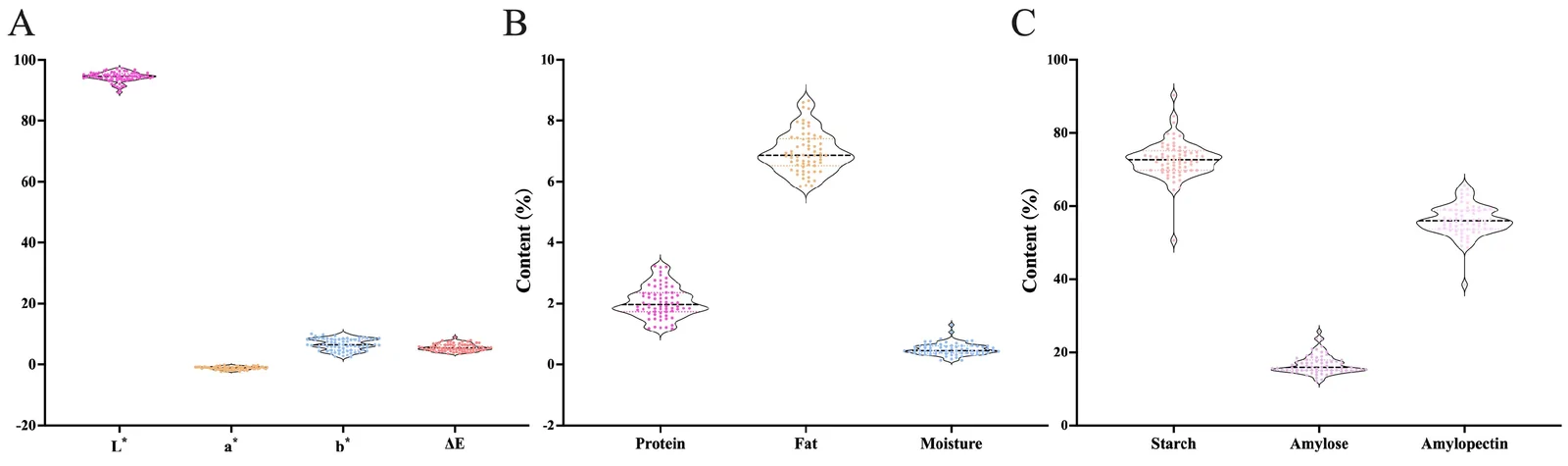
Descriptive statistical analysis results of color parameters (**A**) and major components (**B**,**C**) of cassava flour from 67 varieties. (**A**) L*, a*, b*, and ΔE values; (**B**) protein, fat, and moisture contents; (**C**) starch, amylose, and amylopectin contents.

Color is an important visual characteristic that may influence consumer choices and plays a significant role in assessing food quality [22]. We measured the L*, a*, and b* values of all samples, with the results shown in Figure 1A. The L* value represents brightness and is an important parameter for evaluating color. The higher the L* value, the brighter the color of the sample. a* ranges from red (+) to green (−), and b* ranges from yellow (+) to blue (−) [8]. Cassava roots are predominantly white, whereas yellow varieties are less common but possess higher nutritional value and greater consumer preference. In our study, GS9 showed the highest b* value. The significant difference in b* value mainly reflected the difference in carotenoid (especially β-carotene) content among different varieties, which was a fast and effective indirect index for evaluating the nutritional quality of cassava [23]. Notably, recent metabolomic studies have also revealed that anthocyanins, catechin derivatives, and phenolic acids accumulate at higher levels in yellow-rooted cassava compared with white-rooted ones [24]. Additionally, this indicator is consistent with our visual observations.

### 3.2. Nutrient Content

#### 3.2.1. Protein and Fat

In our study, the protein content of 67 cassava varieties ranged from 1.17% to 3.22% (Figure 1B and Table 1), which is consistent with the findings reported by Borku et al. [25]. The fat content was found to range from 0.13% to 1.30%. These low levels of protein and fat indicate that the dry matter of cassava tuber flour is predominantly composed of carbohydrates, supporting the role of cassava as a staple food that serves as a major energy source for humans.

#### 3.2.2. Starch

Starch is the primary component of cassava tuber dry matter. As shown in Table 1, the highest total starch content of cassava varieties was 90.26%, and the lowest was 50.57%. This variation is due to genetic differences among varieties, consistent with previous reports [26]. Starch is conventionally classified into amylose and amylopectin, and their relative proportions are known to influence functional properties such as gelatinization, retrogradation, and textural characteristics [27]. In our study, amylose content ranged from 16.67% to 25.67%, while amylopectin content ranged from 56.04% to 65.66% (Table 1). Among the varieties analyzed, SC9 exhibited the highest total starch (90.26%) and the highest amylose content (25.67%), whereas GR12 showed the highest amylopectin content (65.66%). Based on general starch chemistry principles, these compositional differences suggest that the tested varieties may differ in their processing suitability. High-amylose starches are generally associated with a higher retrogradation tendency and firmer gel textures, whereas high-amylopectin starches tend to produce softer and more viscous products. However, because the present study focused on compositional analysis rather than direct measurement of functional attributes—such as pasting behavior, swelling power, gel strength, or digestibility—these inferred differences remain hypothetical and require experimental validation. Future work should test the processing performance of these varieties to confirm their suitability for specific uses.

### 3.3. Establishment of NIRS Model

#### 3.3.1. Spectral Characteristics

**Figure 2:**
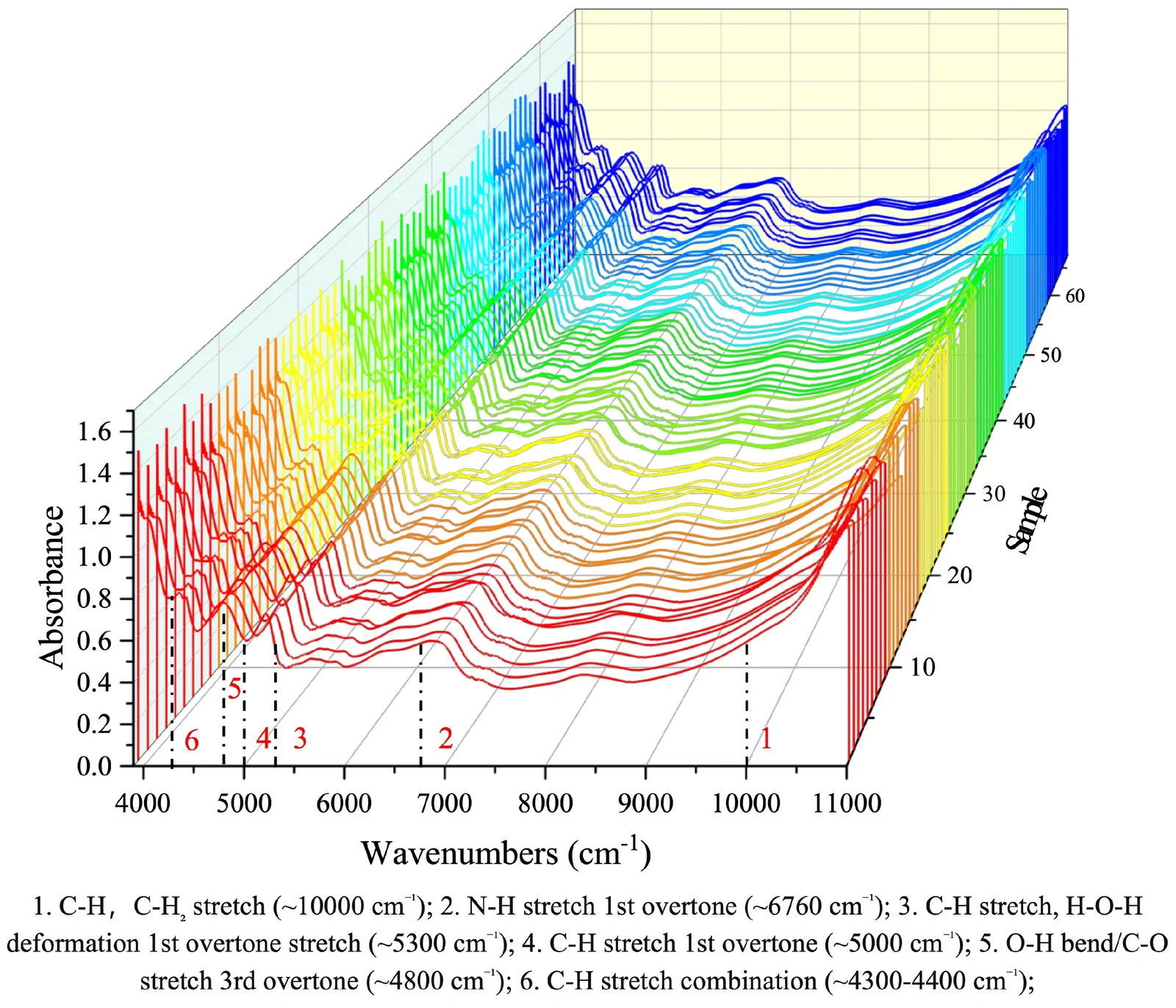
Raw three-dimensional NIRS of the cassava flour samples (n = 67; different cassava flour varieties correspond to different spectral data).

Figure 2 shows the NIRS of all cassava flour samples in the range from 11,000 cm^−1^ to 4000 cm^−1^. The diffuse reflectance spectra of all samples were essentially similar. Cassava consists primarily of carbohydrates, and several major characteristic peaks were also observed in the spectrum. The absorption peaks in the near-infrared region are primarily caused by the stretching vibrations of C–H, O–H, N–H, C=O, and several other chemical bonds [28]. As shown in Figure 2, strong absorption bands were observed at approximately 5000 cm^−1^ and in the 11,000–8000 cm^−1^ region. In contrast, in the wavenumber range of 9000 cm^−1^ to 5000 cm^−1^, the absorbance values generally remained between 0.4 and 0.6, with no significant absorption peaks. These spectral differences may be attributed to the complex composition of cassava, in which different components correspond to different functional groups, and the same group may exhibit varying absorption features depending on its chemical environment. It is worth noting that previous reports have pointed out that the absorption peak near 10,000 cm^−1^ is related to starch content, which is likely to be caused by C–H and C–H_2_ stretching vibrations [29]. Furthermore, the characteristic peak near 6760 cm^−1^ corresponds to the first overtone of N-H stretching vibration, which may indicate the presence of protein-related absorption [28]. The absorption peaks at 5300 cm^−1^ to 5000 cm^−1^ are related to the first overtone of C–H stretching and H–O–H deformation [30]. The absorption peak near 4800 cm^−1^ is related to the third overtone of O–H bending or C–O stretching in carbohydrates [28,31]. The 4400–4300 cm^−1^ region exhibits a dense distribution of C–H bond stretching peaks, which are commonly observed in various organic compounds such as lipids, carbohydrates, and proteins [32,33]. It is worth noting that the prediction performance of the PLS model does not require a single absorption band to be uniquely assigned to the target component; instead, the model exploits the entire spectral region and captures the indirect correlation. Therefore, although we have explained the near-infrared spectral bands, these spectral bands have the characteristics of band overlap and indirect correlation. If accurate analysis of specific components is required, the results may not be accurate enough.

#### 3.3.2. Sample Removal of Outlier Results

**Figure 3:**
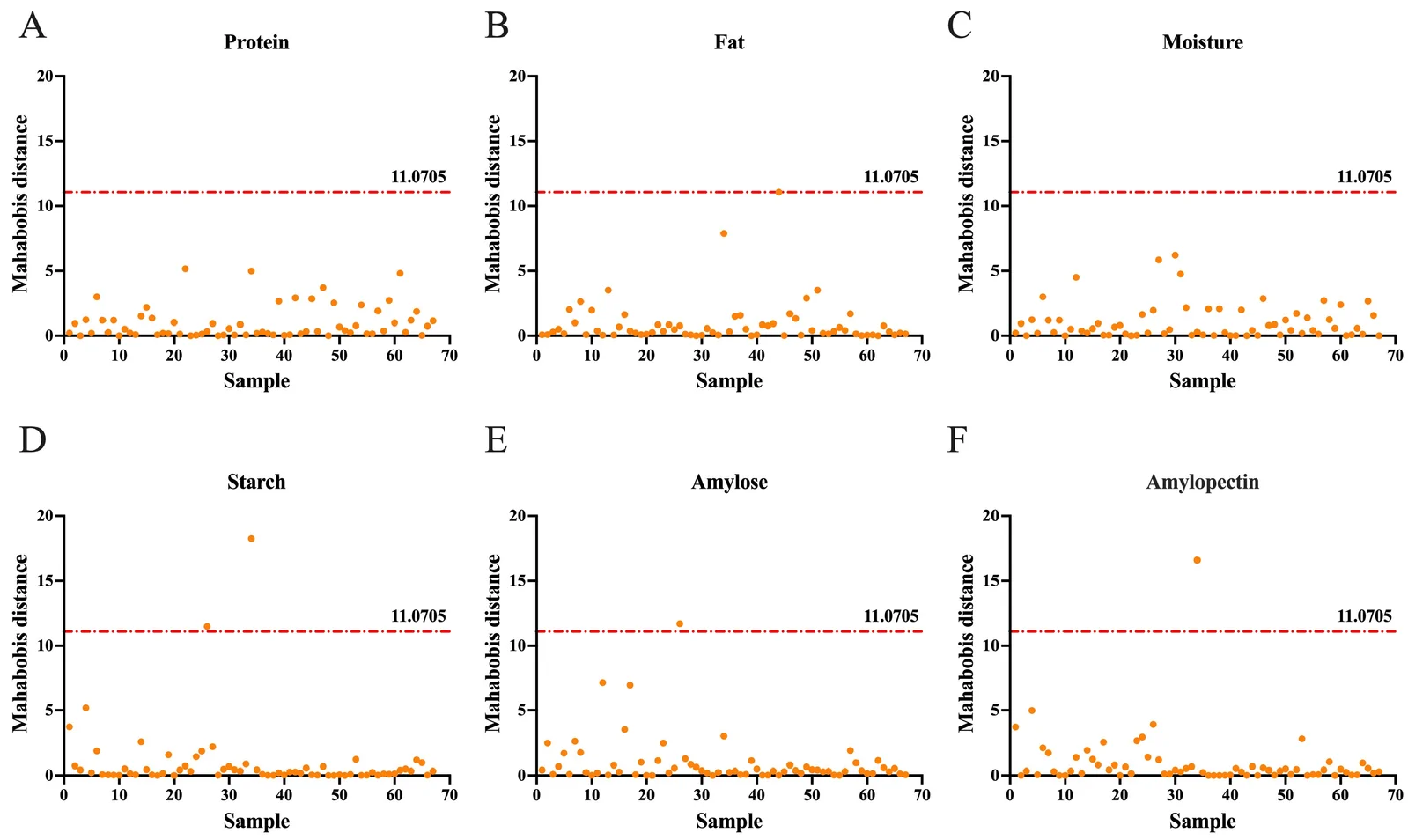
Distribution of Mahalanobis distances of cassava protein (**A**), fat (**B**), moisture (**C**), starch (**D**), amylose (**E**), and amylopectin (**F**).

This study involved 67 cassava flour samples from different varieties, whose inherent compositional variability introduced significant spectral differences in NIRS analysis. To reduce measurement errors and improve predictive accuracy, we identified and removed spectral outliers using Mahalanobis distance combined with chi-square testing (95%). Mahalanobis distance is a multivariate measure of the distance between the sample and the mean value of the spectral matrix. The removal of outliers corrects the deviation of the extreme spectrum, and stabilizes the model without exaggerating the correlation. In this study, the critical Mahalanobis distance threshold was calculated to be 11.07 (Figure 3); any samples exceeding this value were considered outliers and excluded from further analysis. It should be emphasized that the outlier removal was based solely on spectral Mahalanobis distances and was independent of chemical reference values. Accordingly, three samples, SC9, 007 and 021, were excluded. After eliminating outliers, the final calibration set contained 64 samples, which were used to develop prediction models for basic nutritional components. Importantly, the remaining 64 samples still covered almost the entire compositional range of the original 67 samples, indicating that the removal of spectral outliers did not compromise the chemical diversity of the calibration set.

#### 3.3.3. Determination of the Optimal Spectral Preprocessing Method

**Figure 4:**
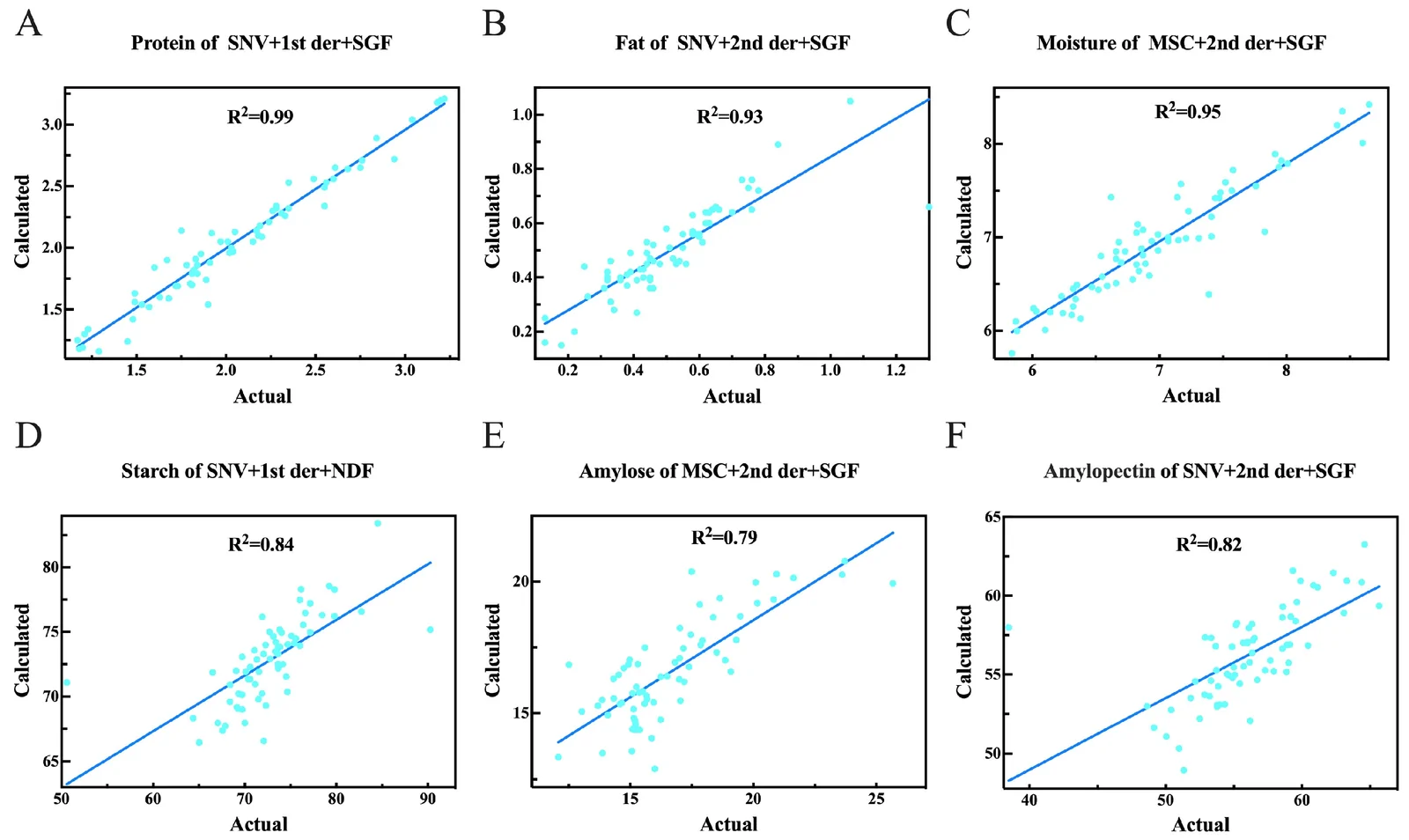
Regression analysis plots between NIRS-predicted and chemically analyzed values for calibration and cross-validation sets. Regression analysis plots for protein (**A**); regression analysis plots for fat (**B**); regression analysis plots for moisture (**C**); regression analysis plots for starch (**D**); regression analysis plots for amylose (**E**); regression analysis plots for amylopectin (**F**).

During near-infrared spectral analysis, a single processing method often fails to effectively and accurately predict the model. Therefore, spectral preprocessing is typically performed by selecting several individual or combined methods to enhance the signal-to-noise ratio, eliminate interfering information, and ultimately establish stable and reliable models [34]. We use correlation coefficients, standard errors, and residual prediction deviation values to determine the optimal spectral processing method. As shown in Table 2, the protein prediction model exhibited the best performance, with the optimal pretreatment method being MSC+1st der+SGF ($R_{c}^{2}$ = 0.99, $R_{cv}^{2}$ = 0.87, RPD = 10.26). The optimal pretreatment method for moisture and amylose was MSC+2nd der+SGF, while that for fat and amylopectin was SNV+2nd der+SGF. For starch, the optimal pretreatment method was SNV+1st der+NDF. The predictive model for amylose content was relatively low ($R_{c}^{2}$ = 0.79, $R_{cv}^{2}$ = 0.34, RPD = 2.21), which may be attributed to its genetic diversity [35]. Furthermore, Figure 4 shows a regression analysis plot comparing NIRS predictions with chemical analysis results, providing a clearer visual representation of the fit curve obtained using the optimal spectral pre-processing.

#### 3.3.4. Model Construction and Validation

After removing outliers, the NIRS data were processed using the optimal preprocessing method, and the models were validated via cross-validation. Model performance was assessed by RMSEC, $R_{c}^{2}$, RMSECV and $R_{cv}^{2}$. Table 2 summarizes the calibration and cross-validation performance of the six NIRS models. Protein and moisture achieved excellent calibration ($R_{c}^{2}$ > 0.95) and acceptable cross-validation ($R_{cv}^{2}$ > 0.83), while starch, amylose, amylopectin, and fat showed good calibration fit ($R_{c}^{2}$ = 0.79–0.93) but poor cross-validation ($R_{cv}^{2}$ = 0.16–0.36), indicating overfitting. The predictive performance varied considerably across different constituents, which may be attributed to differences in the compositional profiles among cassava varieties. These variations could influence the absorption and scattering of NIRS and thus affect model accuracy. This finding is consistent with the results reported by Zhu et al., who utilized NIRS to predict nutritional indicators in different pea varieties [18]. Notably, unlike previous failed attempts using fresh cassava tubers due to poor light penetration use of dried whole flour enabled more accurate model development.

The excellent prediction performance of moisture and protein can be largely attributed to their favorable concentration ranges and unique spectral characteristics. The moisture content in cassava flour ranged from 5.84% to 8.65%, with an average of about 7.0%. Although its absolute range is relatively narrow, the O–H stretching frequency doubling of water produces a strong and characteristic absorption band near 6900–5200 cm^−1^, which is well separated from the absorption peaks of most carbohydrates. Although the protein content is low (1.17–3.22%), its N–H stretching frequency doubling falls in a spectral region that is relatively unaffected by the overlapping signal of starch and water intensity (about 6760 cm^−1^ and 4900–4500 cm^−1^ regions). In addition, the total protein content in cassava flour is mainly determined by the proportion of non-starch components, and its intra-matrix variability may be less than starch structural parameters. In contrast, the fat content is extremely low (0.13–1.30%), and its C–H stretching frequency doubling is severely masked by the dominant starch matrix that accounts for 50–90% of the sample composition.

Total starch is the main spectral contributor in cassava flour, but its concentration range (50.57–90.26%) is extremely wide. Due to the scattering effect and the saturation of frequency doubling absorption, the relationship between total starch and NIRS is nonlinear at high concentrations. In addition, the content of amylose and amylopectin is controlled by multiple genes. In addition to the total content, there is considerable structural diversity (such as chain length distribution and branch chain length). These factors may affect their spectral characteristics, but they are not reflected in the reference chemical values. This structural variation introduces additional noise into the calibration, which further reduces the model performance. The models for total starch, amylose, and amylopectin exhibited a similar fitting performance, which is consistent with their intrinsic chemical relationship as interrelated components of cassava starch. Notably, a considerable gap was observed between calibration and cross-validation performances for fat (Rc^2^ = 0.93 vs. $R_{cv}^{2}$ = 0.16), amylopectin ($R_{c}^{2}$ = 0.82 vs. $R_{cv}^{2}$ = 0.19), and starch ($R_{c}^{2}$ = 0.84 vs. $R_{cv}^{2}$ = 0.36), indicating a risk of overfitting. This difference may be due to the limited sample size (n = 67) and the high dimensionality of NIRS data, which makes the PLS model more inclined to capture noise rather than real chemical signals. The relatively low fat concentration (0.13–1.30%) and the special spectral characteristics of amylose and amylopectin were partially masked by the main starch matrix. The structure and genetic complexity of starch components are controlled by multiple genes and exhibit considerable variety diversity. These findings underscore the importance of critically evaluating calibration and cross-validation statistics, rather than relying solely on calibration performance.

Based on the above statistics, the six models fall into three distinct categories regarding their practical utility. Protein and moisture are the only two models with acceptable cross-validation performance and RPD values above 4.0, supporting their use in preliminary quantitative analysis. Starch, amylose, and amylopectin exhibit moderate calibration performance but poor cross-validation generalization, indicating that the apparent calibration fit is largely driven by overfitting to the specific calibration set; these models are only suitable for preliminary screening. The fat model, despite a high Rc^2^ (0.93), has a negligible $R_{cv}^{2}$ (0.16) and an RPD of 1.08, which unequivocally indicates severe overfitting; this model should not be used for prediction in any form.

### 3.4. Multivariate Analysis of Quality Indicators and Varietal Differentiation

#### 3.4.1. Correlation Analysis

**Figure 5:**
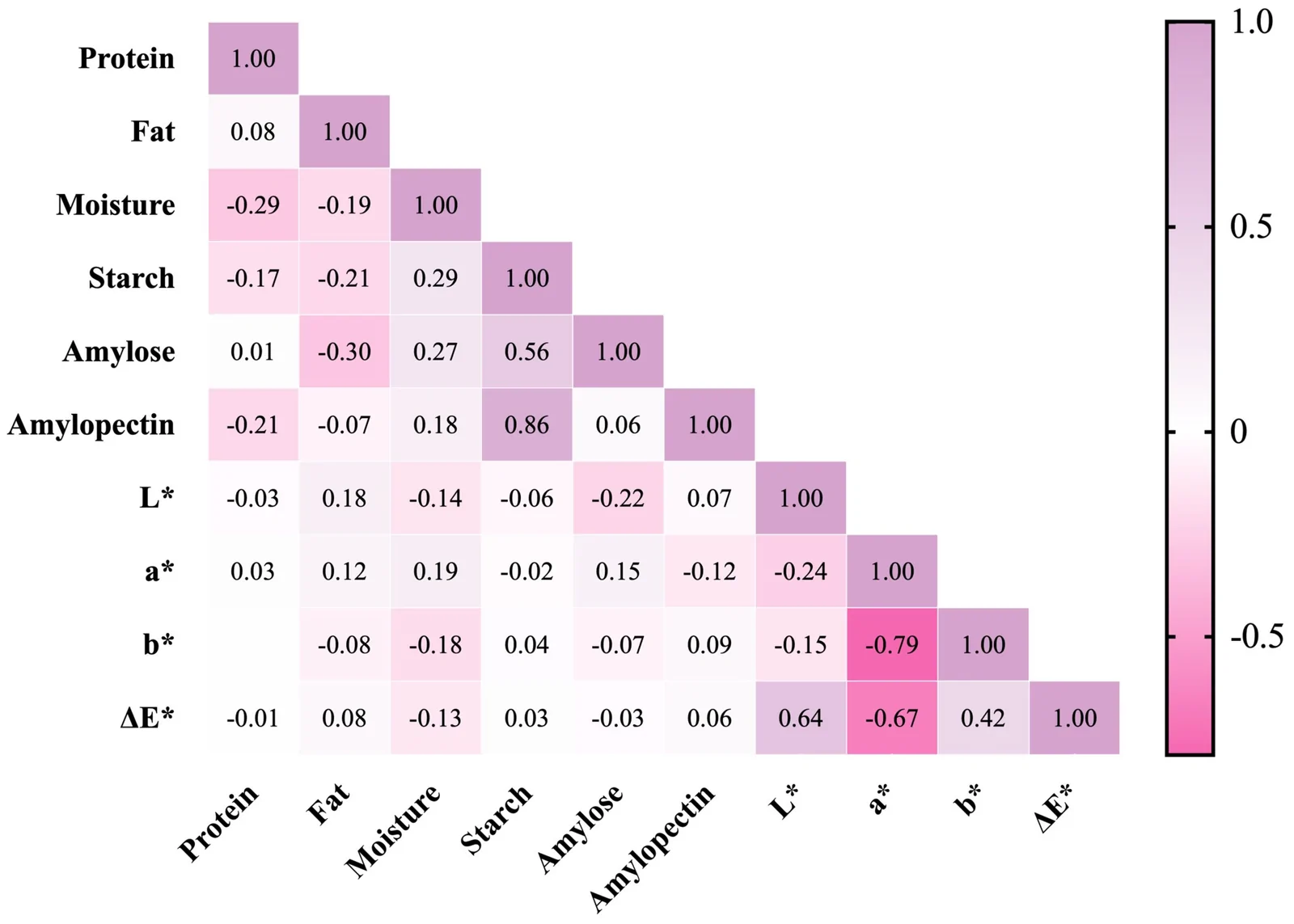
The correlation analysis based on the color characteristics and nutritional quality of cassava flour samples.

To determine the relationship between the nutritional indicators, we used Pearson’s correlation analysis to process the data (Figure 5). We found a positive correlation between amylose, amylopectin, and total starch content. This result is consistent with the general understanding that total starch consists of amylose and amylopectin. Regarding basic nutritional indicators, protein and fat exhibit a positive correlation with amylose, while showing a negative correlation with all other components. In terms of chromaticity index, a* and b* exhibit a significant negative correlation, with b* typically indicating the richness of components abundant in cassava. Our results showed that the b* value was only positively correlated with starch and amylopectin content.

#### 3.4.2. Principal Component Analysis (PCA)

**Figure 6:**
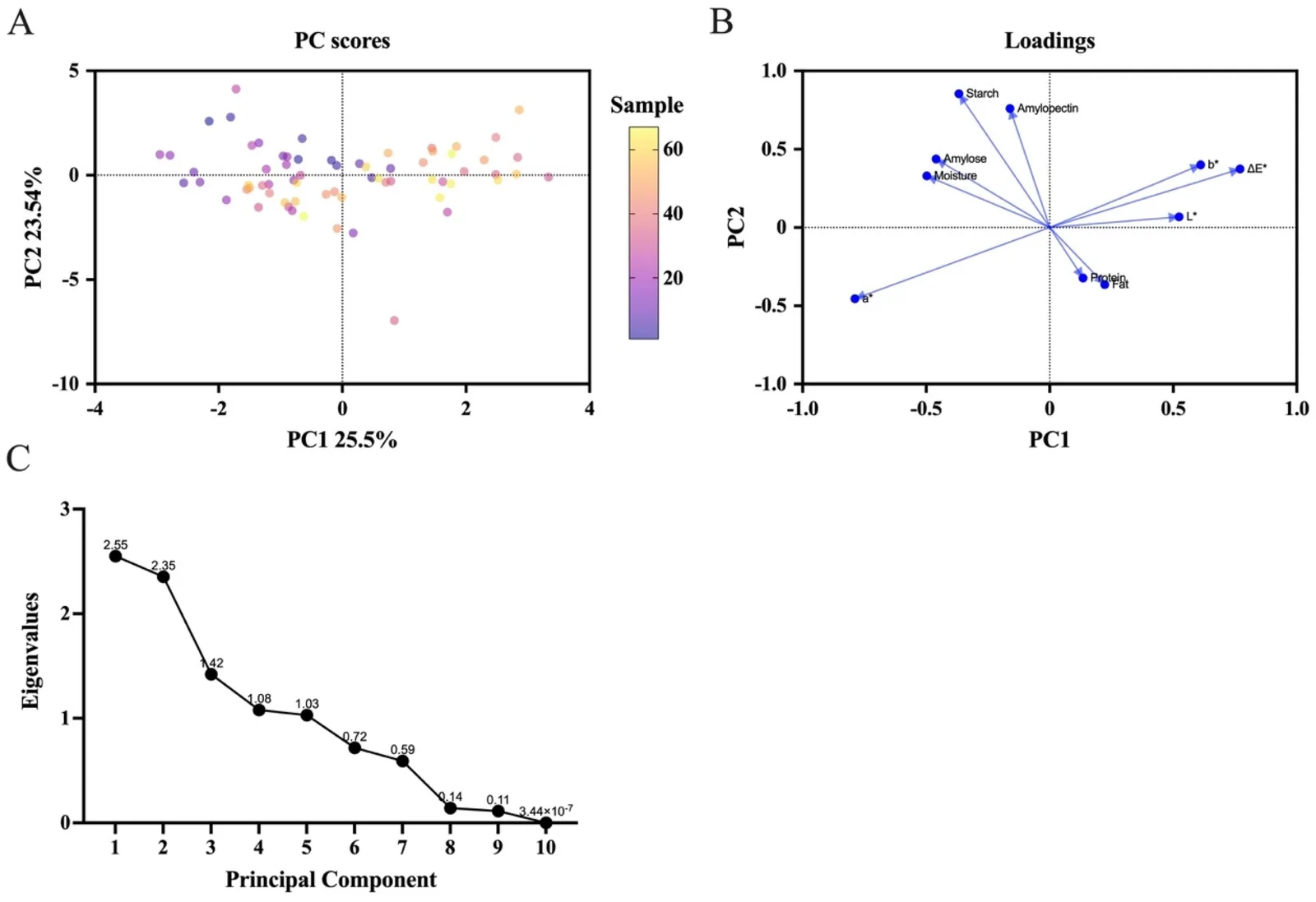
Principal component analysis based on unique nutrient composition of cassava flour samples score plot (**A**); loading plot (**B**); and scree plot (**C**).

To more accurately assess the relationships among the indicators, we conducted further analysis using PCA. The loading plot illustrates the correlations between principal components and variables, where loadings closer to 1 or −1 indicate a greater contribution to the principal component [36]. As shown in Figure 6A, most cassava samples clustered together, with only a few exhibiting significant deviations (004 and 007). We found that 004 has relatively high protein content and relatively low amylose content; 007 has high protein content but low total starch content. Both samples exhibited distinct characteristics and significant variability, resulting in their separation from the remaining samples. As shown in Figure 6A,B, PC1 accounted for 25.50% of the total variance and was predominantly loaded by the color-related parameters L*, a*, b* and ΔE. The loading pattern indicates that b* (yellowness) contributed substantially to PC1, which is consistent with the known visual distinction between white- and yellow-fleshed cassava varieties. However, this observation reflects a statistical association rather than a biological definition of PC1 as a color dimension. PC2 explains 23.54% of the variance, with its loadings concentrated primarily on total starch and amylopectin. Varieties with higher total starch content (such as SC9) tend to have higher PC2 scores. This loading pattern suggests a correlation between PC2 and starch accumulation; however, this should be understood as a data-driven correlation rather than a direct measure of “starch quality” or “accumulation capacity” per se. Additionally, a score plot is commonly used to compare differences among samples and their distribution in the principal component analysis. In our data, the closer the different cassava varieties are located, the higher their similarity. As shown in Figure 6C, the cumulative contribution of the top five principal components in cassava reaches 84.35%, with all eigenvalues exceeding 1. The first five PCs cumulatively explained 84.35% of the total variance, with eigenvalues exceeding 1. PC1 was dominated by color parameters (L*, a*, b* and ΔE; eigenvalue = 2.55), and PC2 was dominated by total starch and amylopectin (eigenvalue = 2.35).

#### 3.4.3. Limitations and Future Perspectives

While this study verified the feasibility of NIRS technology for simultaneous detection of multiple indicators of cassava flour, there are also some methodological limitations. Firstly, it should be noted that the selection of spectral pretreatment methods and the optimal number of latent variables was performed using the full dataset prior to cross-validation, rather than being nested within each cross-validation fold. Consequently, the reported cross-validation performance may be somewhat optimistic due to selection bias, and the model performance should be interpreted with this limitation in mind. Secondly, the model has good calibration for protein (RPD = 10.26) and moisture (RPD = 4.36) (which can be used for routine preliminary quantitative analysis), but the prediction accuracy of fat, amylose and amylopectin is low (RPD is 2.49, 2.21 and 2.36, respectively), and the fat model has serious overfitting (RPD = 1.08, $R_{cv}^{2}$ = 0.16), making it only suitable as a preliminary screening tool and not recommended for predictive applications. Third, although the difference in composition suggested the potential use of varieties such as SC9 and GR12 in different processing, this study did not directly determine gelatinization characteristics, digestibility or sensory attributes, and the relevant assumptions need to be verified by functional, nutritional and sensory tests. In addition, the model was established based on dry powder. Due to limited light penetration, the applicability to intact fresh tubers was limited, and the samples were only from a single geographical environment and growing season. The robustness of different environments and years has not been verified. Correlation analysis and principal component analysis are only for exploratory use and cannot support decision-making.

To improve the robustness and generalizability of the current models, several strategies could be pursued in future work: (i) expanding the sample set to include a wider range of varieties, origins, and growing seasons to increase the chemical diversity of the calibration set; (ii) exploring advanced chemometric algorithms or deep learning approaches that may be better able to handle nonlinear relationships; and (iii) collecting independent sample sets covering a wider range of varieties, geographical sources, and harvest years for rigorous external validation.

## 4. Conclusions

In this study, 67 cassava germplasm accessions were investigated to evaluate the potential of NIRS combined with PLS for simultaneous multi-indicator quality assessment of cassava flour, and a preliminary screening framework for cassava flour quality traits based on NIRS was established using internal cross-validation. The study demonstrates the potential of NIRS for rapid screening of selected quality parameters in the investigated cassava population, with a particularly promising performance for protein and moisture. The models for fat, starch, amylose and amylopectin require further optimization and independent validation before they can be recommended for routine quantitative applications. The study also confirmed that appropriate spectral preprocessing methods can effectively mitigate non-target interference caused by instrumental noise, thereby improving the robustness of the model. It must be emphasized that the present framework remains exploratory; its deployment in practical breeding selection, raw material grading or process control requires external validation with a broader range of varieties and environmental conditions. Overall, despite the many limitations in modeling accuracy and scope of application, this work has generated fundamental technical parameters for high-throughput quality evaluation of cassava germplasm and offers a reference for the future establishment of standardized and generalizable near-infrared rapid detection systems.

## Figures and Tables

**Table 1:** The content of basic nutrients in cassava flour using the chemical method.

Nutrients	Mean	Max	Min
L*	94.32 ± 2.10	97.76 ± 0.37	87.91 ± 0.89
a*	−1.20 ± 0.71	0.16 ± 0.15	−2.58 ± 0.14
b*	6.49 ± 2.64	11.46 ± 0.24	2.06 ± 0.33
ΔE	5.62 ± 1.61	9.40 ± 0.32	2.64 ± 0.19
Protein (%)	2.05 ± 0.52	3.22 ± 0.02	1.17 ± 0.03
Fat (%)	0.50 ± 0.20	1.30 ± 0.03	0.13 ± 0.01
Moisture (%)	6.97 ± 0.68	8.65 ± 0.33	5.84 ± 0.21
Starch (%)	72.71 ± 5.17	90.26 ± 0.66	50.57 ± 0.21
Amylose (%)	16.67 ± 2.64	25.67 ± 0.53	12.08 ± 0.24
Amylopectin (%)	56.04 ± 4.31	65.66 ± 0.77	38.48 ± 0.36

**Table 2:** PLS model calibration and cross-validation results for nutritional content in cassava flour. MSC, multiplicative signal correction; SNV, standard normal variate; 1st Der, first derivative; 2nd Der, second derivative; SGF, Savitzky–Golay filter; NDF, Norris derivative filter; LVs, best latent variables; $R_{c}^{2}$, coefficient of determination for calibration; RMSEC, root mean square error of calibration; $R_{cv}^{2}$, cross-validation coefficient of determination; RMSECV, cross-validation root mean square error; RPD, residual predictive deviation.

Composition	Pretreatment Method	LVs	Calibration Set		Cross-Validation Set		RPD
			$R_{c}^{2}$	RMSEC	$R_{cv}^{2}$	RMSECV	
Protein	MSC+1st der+SGF	10	0.99	0.07	0.87	0.26	10.26
Fat	SNV+2nd der+SGF	8	0.93	0.06	0.16	0.21	1.08
Moisture	MSC+2nd der+SGF	6	0.95	0.20	0.83	0.36	4.36
Starch	SNV+1st der+NDF	8	0.84	2.11	0.36	4.03	2.49
Amylose	MSC+2nd der+SGF	8	0.79	1.45	0.34	2.62	2.21
Amylopectin	SNV+2nd der+SGF	9	0.82	2.03	0.19	4.48	2.36

**Note**: Protein and moisture models can be used for preliminary quantitative analysis; the fat model is not recommended; starch, amylose and amylopectin are recommended for preliminary screening.

## Data Availability

The original contributions presented in this study are included in the article/Supplementary Materials. Further inquiries can be directed to the corresponding author.

## Supplementary Materials

The following supporting information can be downloaded at: https://www.mdpi.com/article/10.3390/foods15173139/s1, Table S1. Information of 67 Cassava samples. Table S2. Effects of different spectral pretreatment on the PLS model of cassava protein. Table S3. Effects of different spectral pretreatment on the PLS model of cassava fat. Table S4. Effects of different spectral pretreatment on the PLS model of cassava moisture. Table S5. Effects of different spectral pretreatment on the PLS model of cassava starch. Table S6. Effects of different spectral pretreatment on the PLS model of cassava amylose. Table S7. Effects of different spectral pretreatment on the PLS model of cassava amylopectin.
