# Supplementary material for: Establishment of a Near-Infrared Spectroscopy-Based Screening Framework for Key Quality Indicators of Cassava Varieties
Source: Foods. 2026 Sep 4;15(17):3139. doi: 10.3390/foods15173139 (PMC13564516; doi:10.3390/foods15173139)
Supplement: Supplementary file 1 [file foods-15-03139-s001.zip › foods-4494156-supplementary.pdf]

Supplementary information

Establishment of a Near-Infrared Spectroscopy-Based Screening Framework for Key Quality Indicators of Cassava Varieties

Supplementary Table S1

Table S1. Information of 67 Cassava samples.

Number	Sample name	Abbreviation
1	Guire No. 4	GR4
2	Guire No. 10	GR10
3	Guire No. 11	GR11
4	Guire No. 12	GR12
5	Guire No. 13	GR13
6	Guishu No. 1	GS1
7	Guishu No. 2	GS2
8	Guishu No. 3	GS3
9	Guishu No. 4	GS4
10	Guishu No. 5	GS5
11	Guishu No. 8	GS8
12	Guishu No. 9	GS9
13	Guishu No. 12	GS12
14	Guishu No. 1505	GS1505
15	Guishu No. 1508	GS1508
16	V4-8	V4-8
17	V4-19	V4-19
18	Q13-43	Q13-43
19	TCGRI008	TCGRI008
20	TCGRI018	TCGRI018
21	TCGRI019	TCGRI019
22	TCGRI028	TCGRI028
23	TCGRI029	TCGRI029
24	TCGRI031	TCGRI031
25	South China No. 101	SC101
26	South China No. 9	SC9

27	South China No. 13	SC13
28	South China No. 14	SC14
29	001	001
30	002	002
31	004	004
32	005	005
33	006	006
34	007	007
35	009	009
36	010	010
37	011	011
38	012	012
39	013	013
40	014	014
41	015	015
42	016	016
43	017	017
44	021	021
45	022	022
46	023	023
47	024	024
48	025	025
49	026	026
50	027	027
51	030	030
52	032	032
53	033	033
54	034	034
55	035	035

56	036	036
57	037	037
58	BRA	BRA
59	Bayin No. 1	BY1
60	Bayin No. 2	BY2
61	Columbia No. 1	CLB1
62	Columbia No. 2	CLB2
63	Kamyin No.3	KY3
64	Limin	LM
65	Nanzhi 199	NZ199
66	Shatian	ST
67	Tianyinong	TYN

Supplementary Table S2

Table S2. Effects of different spectral pretreatment on the PLS model of cassava protein.

Composition	Pretreatment method	LVs	Calibration set		cross-validation set		RPD
			R_c^2	RMSEC	R_{cv}^2	RMSECV	
Protein	Undecided + 1st der + SGF	7	0.87	0.26	0.77	0.35	2.79
	Undecided + 2nd der + SGF	4	0.69	0.39	0.42	0.50	1.80
	Undecided + 1st der + NDF	7	0.93	0.19	0.85	0.29	3.84
	Undecided + 2nd der + NDF	9	0.97	0.13	0.93	0.20	5.63
	MSC + 1st der + SGF	10	0.99	0.07	0.87	0.26	10.26
	MSC + 2nd der + SGF	5	0.81	0.31	0.43	0.50	2.27
	MSC + 1st der + NDF	8	0.95	0.16	0.91	0.22	4.67
	MSC + 2nd der + NDF	10	0.99	0.09	0.94	0.19	8.16
	SNV + 1st der + SGF	10	0.99	0.07	0.87	0.26	10.05
	SNV + 2nd der + SGF	2	0.44	0.47	0.30	0.50	1.33
	SNV + 1st der + NDF	10	0.98	0.10	0.92	0.21	7.24
	SNV + 2nd der + NDF	10	0.98	0.10	0.94	0.19	7.60

Supplementary Table S3

Table S3. Effects of different spectral pretreatment on the PLS model of cassava fat.

Composition	Pretreatment method	LVs	Calibration set		cross-validation set		RPD
			R_c^2	RMSEC	R_{cv}^2	RMSECV	
Fat	Undecided + 1st der + SGF	8	0.87	0.09	0.15	0.20	2.75
	Undecided + 2nd der + SGF	8	0.93	0.64	0.15	0.22	3.77
	Undecided + 1st der + NDF	8	0.64	0.13	0.14	0.20	1.67
	Undecided + 2nd der + NDF	8	0.74	0.12	0.34	0.17	1.95
	MSC + 1st der + SGF	8	0.86	0.08	0.13	0.20	2.69
	MSC + 2nd der + SGF	8	0.93	0.06	0.16	0.21	3.83
	MSC + 1st der + NDF	8	0.65	0.13	0.17	0.19	1.68
	MSC + 2nd der + NDF	8	0.75	0.11	0.40	0.18	2.01
	SNV + 1st der + SGF	8	0.85	0.09	0.13	0.21	2.58
	SNV + 2nd der + SGF	8	0.93	0.06	0.16	0.21	3.89
	SNV + 1st der + NDF	8	0.65	0.13	0.17	0.19	1.68
	SNV + 2nd der + NDF	8	0.75	0.11	0.39	0.17	2.01

Supplementary Table S4

Table S4. Effects of different spectral pretreatment on the PLS model of cassava moisture.

**Composition**	**Pretreatment method**	**LVs**	**Calibration set**		**cross-validation set**		**RPD**
			R_c^2	**RMSEC**	R_{cv}^2	**RMSECV**	
Moisture	Undecided + 1st der + SGF	5	0.91	0.28	0.86	0.35	3.26
	Undecided + 2nd der + SGF	7	0.89	0.30	0.67	0.51	3.08
	Undecided + 1st der + NDF	8	0.90	0.29	0.86	0.34	3.15
	Undecided + 2nd der + NDF	8	0.90	0.28	0.86	0.34	3.21
	MSC + 1st der + SGF	2	0.86	0.32	0.84	0.35	2.66
	MSC + 2nd der + SGF	6	0.95	0.20	0.83	0.36	4.36
	MSC + 1st der + NDF	3	0.88	0.30	0.84	0.34	2.87
	MSC + 2nd der + NDF	3	0.88	0.30	0.84	0.34	2.87
	SNV + 1st der + SGF	2	0.87	0.31	0.84	0.35	2.72
	SNV + 2nd der + SGF	6	0.95	0.20	0.82	0.40	4.30
	SNV + 1st der + NDF	3	0.88	0.30	0.84	0.34	2.90
	SNV + 2nd der + NDF	3	0.88	0.30	0.84	0.34	2.89

Supplementary Table S5

Table S5. Effects of different spectral pretreatment on the PLS model of cassava starch.

Composition	Pretreatment method	LVs	Calibration set		cross-validation set		RPD
			R_c^2	RMSEC	R_{cv}^2	RMSECV	
Starch	Undecided + 1st der + SGF	8	0.82	2.21	0.41	3.83	2.36
	Undecided + 2nd der + SGF	8	0.76	2.51	0.47	3.58	2.05
	Undecided + 1st der + NDF	8	0.76	2.51	0.47	3.58	2.05
	Undecided + 2nd der + NDF	8	0.76	2.51	0.45	3.65	2.05
	MSC + 1st der + SGF	8	0.82	2.21	0.39	3.97	2.37
	MSC + 2nd der + SGF	8	0.83	2.11	0.36	4.08	2.49
	MSC + 1st der + NDF	8	0.77	2.48	0.48	3.55	2.08
	MSC + 2nd der + NDF	8	0.77	2.47	0.47	3.59	2.09
	SNV + 1st der + SGF	8	0.82	2.19	0.46	3.72	2.38
	SNV + 2nd der + SGF	8	0.77	2.49	0.47	3.58	2.07
	SNV + 1st der + NDF	8	0.84	2.11	0.38	4.03	2.49
	SNV + 2nd der + NDF	8	0.77	2.48	0.46	3.6	2.08

Supplementary Table S6

Table S6. Effects of different spectral pretreatment on the PLS model of cassava amylose.

Composition	Pretreatment method	LVs	Calibration set		cross-validation set		RPD
			R_c^2	RMSEC	R_{cv}^2	RMSECV	
Amylose	Undecided + 1st der + SGF	2	0.78	1.50	0.26	2.91	2.11
	Undecided + 2nd der + SGF	8	0.79	1.46	0.30	2.73	2.19
	Undecided + 1st der + NDF	8	0.71	1.66	0.34	2.47	1.88
	Undecided + 2nd der + NDF	8	0.73	1.64	0.37	2.39	1.91
	MSC + 1st der + SGF	8	0.79	1.48	0.30	2.74	2.16
	MSC + 2nd der + SGF	8	0.79	1.45	0.34	2.62	2.21
	MSC + 1st der + NDF	8	0.71	1.67	0.37	2.40	1.87
	MSC + 2nd der + NDF	8	0.73	1.64	0.40	2.33	1.91
	SNV + 1st der + SGF	8	0.78	1.50	0.29	2.80	2.13
	SNV + 2nd der + SGF	8	0.79	1.45	0.33	2.64	2.20
	SNV + 1st der + NDF	8	0.71	1.67	0.36	2.41	1.87
	SNV + 2nd der + NDF	8	0.72	1.64	0.39	2.33	1.91

Supplementary Table S7

Table S7. Effects of different spectral pretreatment on the PLS model of cassava amylopectin.

Composition	Pretreatment method	LVs	Calibration set		cross-validation set		RPD
			R_c^2	RMSEC	R_{cv}^2	RMSECV	
Amylopectin	Undecided + 1st der + SGF	9	0.81	2.11	0.15	4.77	2.27
	Undecided + 2nd der + SGF	9	0.82	2.03	0.22	4.33	2.36
	Undecided + 1st der + NDF	9	0.73	2.42	0.31	3.91	1.94
	Undecided + 2nd der + NDF	9	0.74	2.41	0.29	3.81	1.95
	MSC + 1st der + SGF	9	0.79	2.16	0.01	5.28	2.21
	MSC + 2nd der + SGF	9	0.82	2.04	0.19	4.47	2.36
	MSC + 1st der + NDF	9	0.73	2.44	0.28	3.98	1.92
	MSC + 2nd der + NDF	9	0.73	2.43	0.24	4.01	1.93
	SNV + 1st der + SGF	9	0.81	2.08	0.07	5.07	2.30
	SNV + 2nd der + SGF	9	0.82	2.03	0.19	4.48	2.36
	SNV + 1st der + NDF	9	0.73	2.44	0.28	3.96	1.92
	SNV + 2nd der + NDF	9	0.73	2.42	0.26	3.98	1.94
